# Supplementary material for: Tumor suppression in basal keratinocytes via dual non-cell-autonomous functions of a Na,K-ATPase beta subunit
Source: eLife. 2016 May 30;5:e14277. doi: 10.7554/eLife.14277 (PMC4973367; doi:10.7554/eLife.14277)
Supplement: Figure 3—figure supplement 1—source data 1. — In addition to atp1b1a (in red), the exons of the other 42 genes in the region were sequenced during this study. Furthermore, the exons or cDNAs of 10 of these 42 genes had already been sequenced by Webb et al. (2008). Apart from the described nonsense mutation in atp1b1a, no mutagenic lesions were found. DOI: http://dx.doi.org/10.7554/eLife.14277.012 [file elife-14277-fig3-figsupp1-data1.docx]

| Gene | location | sequenced in this study | sequenced in Webb et al., 2008 |
| --- | --- | --- | --- |
| *cep63* | 27,906,072-27,934,909 | x |  |
| *amotl2a* | 28,001,382-28,010,531 | x |  |
| *sap130a* | 28,027,829-28,059,256 | x |  |
| *myo7b* | 28,061,417-28,121,143 | x |  |
| *si:ch73-194h10.3* | 28,124,822-28,127,434 | x |  |
| *gpr17* | 28,133,832-28,134,851 | x |  |
| *inpp5d* | 28,150,255-28,201,941 | x |  |
| *smx5* | 28,214,723-28,217,816 | x |  |
| *si:ch73-14h10.2* | 28,218,412-28,219,711 | x |  |
| *Bcl6a* | 28,225,926-28,232,031 | x |  |
| *lpp* | 28,303,552-28,700,109 | x |  |
| *tprg1* | 28,762,382-28,799,758 | x |  |
| *tp63* | 28,805,219-28,923,664 | x |  |
| *tbc1d23* | 28,923,672-28,952,498 | x |  |
| *tomm70a* | 28,955,480-28,971,099 | x |  |
| *Glmnb* | 28,979,952-28,990,268 | x | x |
| *gfi1ab* | 29,019,766-29,095,912 | x | x |
| *evi5b* | 29,019,766-29,095,912 | x |  |
| *rpl5* | 29,096,033-29,106,832 | x | x |
| *fam69ab* | 29,112,381-29,152,902 | x | x |
| *zbtb11* | 29,154,215-29,169,327 | x | x |
| *Dpt* | 29,193,576-29,205,081 | x | x |
| *atp1b1a* | 29,226,831-29,236,926 | x |  |
| *nme7* | 29,236,928-29,297,594 | x | x |
| *zgc:172121* | 29,297,445-29,302,326 | x |  |
| *Bivm* | 29,304,749-29,314,571 | x | x |
| *si:ch211-101h21.5* | 29,314,629-29,321,769 | x |  |
| *tmem131* | 29,322,659-29,386,531 | x |  |
| *actl6a* | 29,396,167-29,405,143 | x |  |
| *mrpl47* | 29,406,993-29,412,017 | x | x |
| *ndufb5* | 29,412,436-29,419,530 | x | x |
| *ups13* | 29,420,425-29,478,647 | x |  |
| *pex5la* | 29,486,372-29,621,708 | x |  |
| *pde6d* | 29,702,931-29,715,434 | x |  |
| *phb2b* | 29,716,568-29,723,729 | x |  |
| *ptmaa* | 29,763,580-29,804,468 | x |  |
| *dlg1* | 29,870,215-30,092,361 | x |  |
| *mfi2* | 30,101,250-30,118,879 | x |  |
| *lrrc40* | 30,123,189-30,131,993 | x |  |
| *lrrc7* | 30,131,084-30,323,275 | x |  |
| *zgc:153311* | 30,504,182-30,606,230 | x |  |
| *wwc3* | 30,504,182-30,606,230 | x |  |
| *lurap1* | 30,643,079-30,671,841 | x |  |
